# Modulation of Phosducin-Like Protein 3 (PhLP3) Levels Promotes Cytoskeletal Remodelling in a MAPK and RhoA-Dependent Manner

**DOI:** 10.1371/journal.pone.0028271

**Published:** 2011-12-09

**Authors:** Nandini V. L. Hayes, Lyne Jossé, C. Mark Smales, Martin J. Carden

**Affiliations:** Centre for Molecular Processing and School of Biosciences, University of Kent, Canterbury, Kent, United Kingdom; Stanford, United States of America

## Abstract

**Background:**

Phosducin-like protein 3 (PhLP3) forms a ternary complex with the ATP-dependent molecular chaperone CCT and its folding client tubulin. *In vitro* studies suggest PhLP3 plays an inhibitory role in β-tubulin folding while conversely *in vivo* genetic studies suggest PhLP3 is required for the correct folding of β-tubulin. We have a particular interest in the cytoskeleton, its chaperones and their role in determining cellular phenotypes associated with high level recombinant protein expression from mammalian cell expression systems.

**Methodology/Principal Findings:**

As studies into PhLP3 function have been largely carried out in non mammalian systems, we examined the effect of human PhLP3 over-expression and siRNA silencing using a single murine siRNA on both tubulin and actin systems in mammalian Chinese hamster ovary (CHO) cell lines. We show that over-expression of PhLP3 promotes an imbalance of α and β tubulin subunits, microtubule disassembly and cell death. In contrast, β-actin levels are not obviously perturbed. On-the-other-hand, RNA silencing of PhLP3 increases RhoA-dependent actin filament formation and focal adhesion formation and promotes a dramatic elongated fibroblast-like change in morphology. This was accompanied by an increase in phosphorylated MAPK which has been associated with promoting focal adhesion assembly and maturation. Transient overexpression of PhLP3 in knockdown experiments rescues cells from the morphological change observed during PhLP3 silencing but mitosis is perturbed, probably reflecting a tipping back of the balance of PhLP3 levels towards the overexpression state.

**Conclusions:**

Our results support the hypothesis that PhLP3 is important for the maintenance of β-tubulin levels in mammalian cells but also that its modulation can promote actin-based cytoskeletal remodelling by a mechanism linked with MAPK phosphorylation and RhoA-dependent changes. PhLP3 levels in mammalian cells are thus finely poised and represents a novel target for engineering industrially relevant cell lines to evolve lines more suited to suspension or adherent cell growth.

## Introduction

The phosducin-like family of proteins were first identified, through phosducin itself, as proteins proposed to sequester the β and γ subunit dimer of G protein (Gβγ) thereby inhibiting its interaction with the α subunit (Gα) and regulating signalling involving trimeric G-protein coupled receptors in multicellular organisms [1]. More recently it has emerged that the most widely conserved members of this family in eukaryotes, namely excepting mammalian retinal phosducin, act as co-chaperones for the chaperonin containing TCP1 (CCT) [2], [3], [4]. Blaauw *et al.* [5] established three subgroups of phosducin-like proteins on the basis of sequence similarity present from plants to humans via yeast and slime moulds; subtype I, including the original phosducin (Pdc) and its subsequently discovered more generally expressed human relative phosducin-like protein 1 (PhLP1), subtype II represented in humans by PhLP2A and PhLP2B [5], [6] and subtype III which includes human PhLP3 [5].The nomenclature used in this report with regard to the phosducin-like proteins is detailed and clarified in Table 1. From studies to date it appears that PhLP1-3 may all be important as co-chaperones during CCT-assisted protein folding whilst only Pdc and PhLP1 (which have a high affinity for Gβγ) have a role in G protein signalling, phosducin itself being a relatively recent evolutionary product that has lost interaction with CCT (for a review of this area see [7]).

**Table 1:** The nomenclature used for the Phosducin like proteins (PLPs) discussed in this manuscript.

Gene (Human)	Aliases	Protein Name	Term used in this manuscript
PDC	MEKA	Phosducin	PDC
PDCL	PhLP1	Phosducin-like	PhLP1
PDCL2	GCPHLP, PhLP2B	Phosducin-like 2	PhLP2B
PDCL3	PhLP2A, VIAF	Phosducin-like 3	PhLP2A
TXNDC9	PhLP3, APACD	Thioredoxin domain containing 9	PhLP3

A growing body of evidence implicates PhLP3 in the folding pathway of the cytoskeletal components actin and β-tubulin. For example, Stirling and co-workers [4] suggested that PhLP3 (also referred to as APACD or TXNDC9 in mammals, see [4] and Table 1) may be involved during the early stages of actin and βtubulin folding (independent of the prefoldin complex). These *in vitro* studies demonstrated that PhLP3 had a negative effect on actin and tubulin folding, possibly by modulating the ATPase activity of CCT. The yeast subtype III orthologue, confusingly termed Plp1, does not stimulate actin binding by CCT whereas the subtype II orthologue, Plp2, strongly stimulates both binding and folding of actin by CCT [2]. In embryonic nematode worms siRNA silencing of PhLP3 produced defects in astral and spindle pole microtubules and defective cytokinesis [8], mirroring the effect of RNA silencing of two PhLP3 homologues in plant *Arabidopsis thaliana* (PhLP3a and PhLP3b) that resulted in a disrupted microtubule network and subsequent defective cell We have a particular interest in both molecular chaperones and protein folding [9], [10], [11] and the cytoskeleton [12], [13] with respect to their roles in determining cellular phenotypes associated with high level expression of recombinant protein by mammalian cell expression systems e.g. see [14], [15], [16]. Proteomic analyses have also demonstrated a correlation of chaperone and cytoskeletal protein levels with recombinant protein yields in NS0 cells engineered to express an IgG4 monoclonal antibody [17]. Indeed, it is becoming increasingly clear that interactions between the cytoskeleton, the translational machinery, chaperones, protein folding/degradation, and the secretory machinery are coordinated [18], all of which are cellular processes underpinning heterologous protein production during bioprocessing. In this respect there is also evidence that the cytoskeletal apparatus interacts functionally with the translational apparatus and that interference of this interaction leads to a reduction in protein synthesis [19] whilst modulation of mRNA translation is known to influence recombinant protein synthesis in mammalian cells [20], [21], [22]. With regard to CCT, this chaperone has also been linked to translation in various cellular contexts, including nascent chain folding on the ribosome [23], ribosome biogenesis [24], antigen processing [25], and cytoskeletal biogenesis [12], [13], [26]. CCT also shows interactions with components of the translational machinery including elongation and initiation factors [27], [28].

In the bioprocessing industry, the gold standard mammalian cell expression system is the Chinese hamster ovary (CHO) cell [29] which is adapted to grow in suspension growth in large scale bioreactors where the cells have a very different morphology to when grown adherently that presumably requires cytoskeleton remodelling. With our interest in the cytoskeleton and its role in determining cellular phenotypes associated with high level recombinant protein expression from mammalian cell expression systems, and the fact that the majority of studies into PhLP3 function reported to date have been carried out in non-mammalian systems, we set out to investigate the role of PhLP3 in cytoskeleton remodelling in the industrially relevant CHO cell line. Specifically, we investigated the influence of manipulating PhLP3 levels in mammalian cells and show that over-expression of human PhLP3 in a mammalian system disrupts α- and β-tubulin localisation whilst there is an approximate 20% decrease in α-tubulin levels and an approximate 60% increase in β-tubulin level. By contrast, neither actin levels nor assembly appeared to be obviously altered upon over-expression of PhLP3, yet siRNA silencing of PhLP3 promoted the formation of microfilament remodelling and RhoA/ROCK-dependent stress fiber formation which was accompanied by an increase in phosphorylated ERK. Rescue experiments following siRNA prevent the anchorage-dependent morphological changes observed during siRNA silencing but probably reflect a milder overexpression phenotype with inhibited mitosis and promotion of enlarged polyploid nuclei. These findings support the hypothesis that PhLP3 is important for maintaining β-tubulin levels in mammalian cells and that modulation of PhLP3 levels promotes cytoskeletal remodelling in a MAPK and RhoA-dependent manner.

## Results

### Authentic human PhLP3 is expressed in the adherent CHOK1 cell line

**Figure 1:**
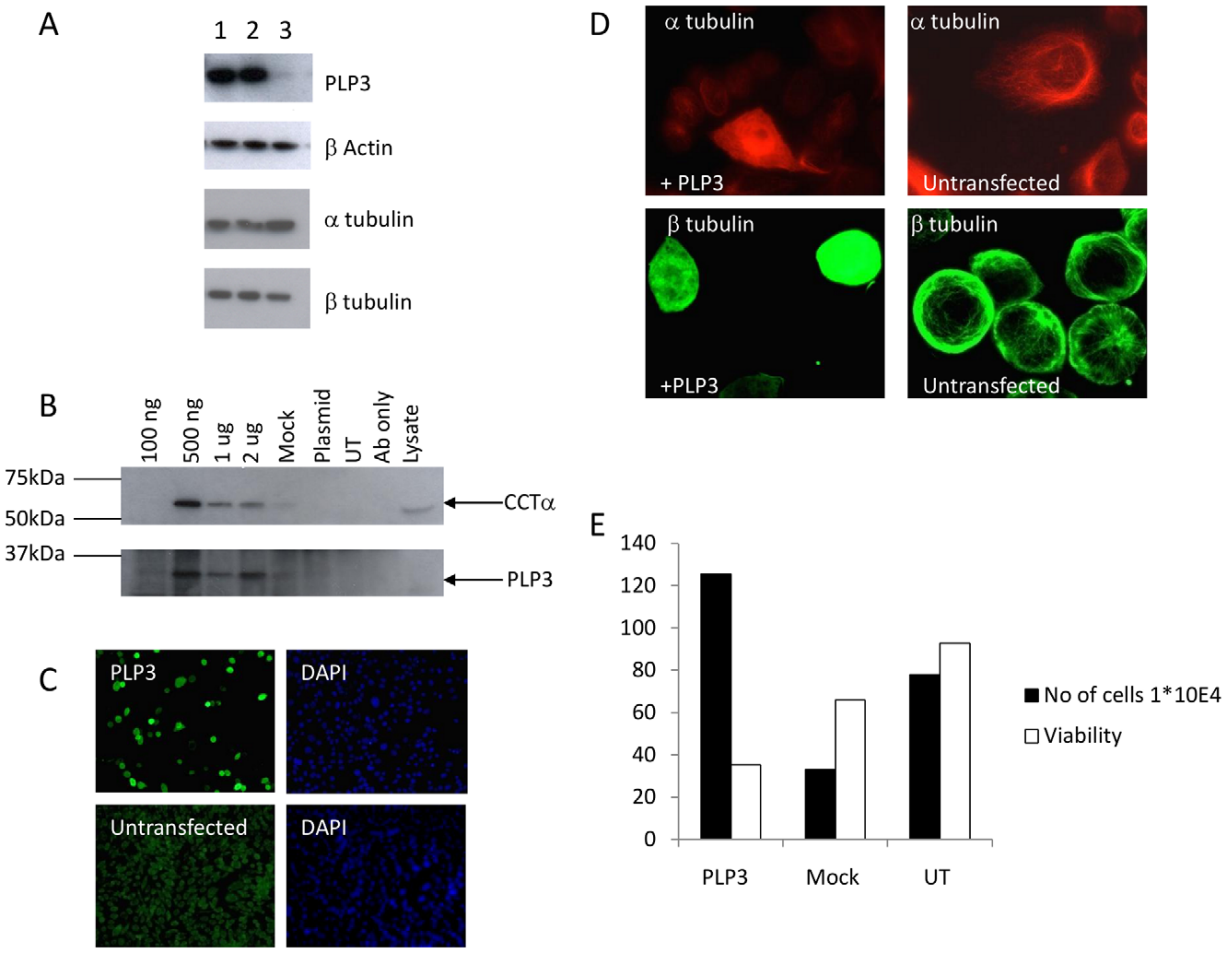
Transient expression of PhLP3 in CHOK1 adherent cells promotes tubulin redistribution, an imbalance of α and β-tubulin subunits and cell death. (A) Anti-PhLP3 antibody detects full length PhLP3 (24 kDa) in CHOK1 adherent cells transiently transfected with human PhLP3 (duplicates lane 1 and 2). Endogenous PhLP3 was detected in the plasmid only control (lane 3). β-actin levels are not altered upon PhLP3 overexpression but this promotes an increase of β-tubulin subunits relative to the control and a decrease in α-tubulin subunits (compare lanes 1 and 2 with lane 3). (B) Immunoprecipitation of CCTα in CHOK1 cells transiently transfected with PhLP3. CCTα (top panel) and PhLP3 (bottom panel) were detected in cell lysates transiently transfected with 500 ng–2 µg PhLP3 plasmid and immunoprecipitated with an anti-PhLP3 antibody, but not in plasmid only, untransfected and antibody only controls. (C) Immunofluorescent detection of PhLP3 in CHOK1 cells transfected with PhLP3. An anti-PhLP3 antibody was used to identify cells overexpressing PhLP3 (green) and DAPI (blue). (D). PhLP3 overexpression alters tubulin distribution. Immunofluorescent staining of PhLP3 transfected CHOK1 cells with either anti-α tubulin(red) or anti-βtubulin(green) specific antibodies detects redistribution of both tubulin subunits compared to untransfected controls. (E) PhLP3 overexpression in adherent CHOK1 cells promotes the release of cells into the supernatant and decreases cell viability compared to mock transfected and untransfected controls. Data calculated as mean +/− SEM of n = 2.

It is reported that recombinant human PhLP3 inhibits CCT mediated folding of actin in a rabbit reticulocyte lysate *in vitro* translation system [4], [30]. However, to our knowledge there have been no reports of the effect of PhLP3 manipulation on the actin/tubulin cytoskeleton in a mammalian cell culture system. Successful over-expression of PhLP3 constructs in our adherently grown CHOK1 model system was initially confirmed by western blotting. Endogenous PhLP3 was detectable at relatively low levels in untransfected cells (track 3, top panel, Figure 1A) but increased greatly when recombinant human PhLP3 was transiently expressed (tracks 1 and 2). Immunofluorescence confirmed this much higher expression of PhLP3 in transfected CHOK1 adherent cells compared with untransfected surrounding cells. Similarly high levels of PhLP3 expression were seen in GFP-positive cells generated by co-transfection used to monitor plasmid uptake by, and transfection of, CHOK1 adherent cells (Figure 1C).

To assess the authenticity of the recombinantly expressed PhLP3 protein, its known activity of binding to CCT [4] was monitored. CHOK1 adherent cells were transfected with a range of DNA amounts (100 ng, 500 ng, 1 µg, 2 µg) and binding of CCT determined in immunoprecipitates generated using antibody to PhLP3. At the lowest level of PhLP3 over-expression (100 ng of plasmid) no CCT subunits were detected in immunoprecipitates, whereas CCTα (and CCTβ data not shown) binding was apparent upon transfection with 500 ng or greater amounts of plasmid (Figure 1B). When higher amounts of plasmid were transfected (1 and 2 µg) there was not an associated increase in PhLP3 over-expression compared to the 500 ng transfection and the amounts of CCTα detected in immunoprecipitation experiments were actually decreased (Figure 1B). A tagged PhLP3 recombinant protein (PhLP3V5 - with a V5 tag at its C-terminus) did not interact with CCTα in this assay (data not shown) and was therefore not used in further work. This finding suggests that modification of the C-terminal end of PhLP3 disrupts its ability to interact with CCT subunits, as observed by Stirling and colleagues [4], though C-terminal myc-tagging does not prevent CCT binding by PhLP1 constructs [31]. Neither actin nor β-tubulin were detected in the immunoprecipitated complexes (data not shown).

### Increasing PhLP3 expression does not appear to perturb actin levels or organisation in CHOK1 adherent cells but results in tubulin re-distribution

Transfection of PhLP3 into CHOK1 cells (or the suspension CHO cell line LB01, data not shown) did not alter the amounts of β-actin detected by immunoblot (Figure 1A, second panel) or in any obvious way the intracellular distribution of actin (not shown). In contrast, over-expression of PhLP3 resulted in a change to the levels of, and ratio of, α- and β-tubulin subunits. There was an approximate 20% decrease in α-tubulin after PhLP3 overexpression mirrored by an approximate 60% increase in β-tubulin subunits (Figure 1A, third and fourth panels), as determined by densitometry analysis of the immunoblots. This change in the ratio of the α- and β-tubulin subunits was associated with a reorganisation of the microtubules (Figure 1D). α-tubulin in particular appeared to be partially redistributed to the nucleoplasm (but not to the nucleolus) (Figure 1D), a most unusual interphase location for tubulin in normal cells. Analysis of the supernatant of adherent CHOK1 cells 48 h post-PhLP3 transfection revealed that there was a 2.5-fold increase in the number of detached cells free in the supernatant (i.e. not adherent) compared to the controls. Moreover, the viability of these cells was just 30% (PhLP3 transfected) compared to 80% in controls (Figure 1E). After 72 h of transfection with 2 µg of plasmid encoding PhLP3 extensive cell death was evident, suggesting toxicity of substantial PhLP3 overexpression levels.

### siRNA silencing of PhLP3 in CHO LB01 suspension cells promotes an elongated morphology, anchorage-dependent growth and an altered actin cytoskeleton

**Figure 2:**
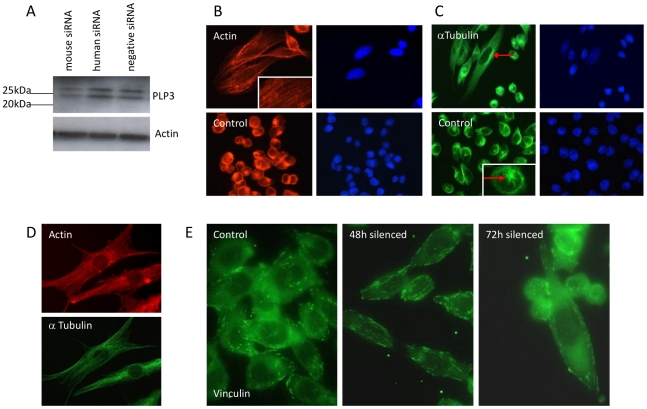
RNAi silencing of PhLP3 in LB01 cells promotes a fibroblast-like morphology and cytoskeletal rearrangement. (A) Immunoblots using anti-PhLP3 of LB01 cells transfected with mouse siRNA shows a decrease in PhLP3 expression compared to human siRNA and a scrambled negative control siRNA (top panel). Actin is used as an internal loading control (bottom panel). Note that two PhLP3 products (bands) are detected in these samples prepared with phosphatase inhibitors while in figure 1A (where they were absent) only one product is detected: PhLP3 is known to be phosphorylated [45] (see also references in the human PhLP3 Unigene entry UniGene Hs.536122) with the phosphoform displaying higher apparent molecular weight on SDS gels than the unphosphorylated form. (B) LB01 cells were transfected with mouse PhLP3 siRNA for 72 h and the cells were fixed and stained for either actin (rhodamine phalloidin) or (C) tubulin (anti-α-tubulin). The microtubule organising center is indicated by a red arrow. (D) NIH 3T3 mouse fibroblast cells were fixed and stained for F-actin filaments (rhodamine phalloidin, red) and microtubules (anti-α-tubulin, green). (E) Focal adhesions observed with anti-vinculin in cells transfected with the control plasmid or siRNA silencing plasmid 48 h and 72 h post transfection.

**Figure 3:**
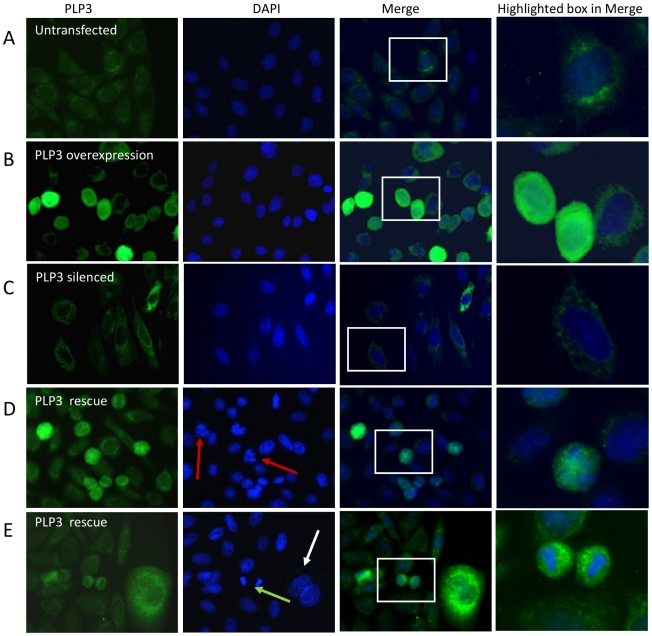
Rescue of PhLP3 silenced CHO LB01 cells promotes aberrant cell division. LB01 cells were silenced and rescued as described in the methods section and then fixed and co-stained for PhLP3 (green) and with the nuclear DAPI stain (blue). PhLP3 overexpression in LB01 cells leads to more ‘rounded’ cells compared to untransfected controls (A v B) whilst siRNA silencing promotes morphological changes including elongated cells and nuclei and adherent growth (A v C). When LB01 cells were co-transfected with mouse PhLP3 siRNA and human PhLP3 DNA (D and E) the elongated phenotype observed in the PhLP3 silenced cells was no longer present and thus the cells were considered ‘rescued’. Co-transfected cells were either in early anaphase (red arrows), late anaphase (green arrow) or exhibited enlarged nuclei (white arrrow).

Previous studies demonstrated that PhLP3 is not essential in *Dictyostelium* [5] and yeast Plp1 (a type III PLP) is not essential, but in *C.elegans* siRNA silencing of PhLP3 resulted in microtubule defects preventing proper cytokinesis in the embryo [8]. To assess the effect of PhLP3 silencing in a cultured mammalian cell system, PhLP3-directed knockdown was conducted using two CHO cell lines, the CHOK1 (adherent) and LB01 (suspension) cell lines. Two siRNAs, one derived from human PhLP3 the other based on mouse PhLP3, were used because they were commercially available and because the annotated CHO genome sequence is not in the public domain. PhLP3 silencing, by the mouse siRNA but not the human siRNA, in CHO LB01 cells was confirmed by qRT-PCR at the mRNA level (Figure S1) and by immunoblotting (Figure 2A) which showed an approximate 65% decrease in endogenous PhLP3 levels consistent with the mRNA data. Together these data, consisting of the qRT-PCR data showing a knockdown of *PhPL3* at the mRNA level and the western data showing a knockdown at the protein level, confirm that the murine siRNA does indeed result in knockdown of the CHO *PhPL3* mRNA. After 72 hours of siRNA silencing treatment a dramatic change in cell phenotype was observed with elongation of both cells and nuclei, the latter being also increased in size (Figure 2, and Figure 3 second panel). This characteristic morphological change compared with untransfected cells was observed only with the mouse siRNA, not with the human siRNA, nor with a scrambled sequence (negative control) siRNA.

The elongated morphology of the cells upon knockdown suggested cytoskeletal changes, most likely in actin assembly as stress fibres. The presence of extensive stress fiber formation was indeed confirmed using phalloidin (Figure 2B). There were no obvious increases in lamellipodia or membrane ruffles. There was an accompanying reorganisation of the microtubule network, from a radiating pattern emanating from a distinct microtubule organising centre (MTOC) to more extensive elongated parallel microtubule arrays (Figure 2C) characteristically observed in flat, adherent fibroblast cells with well-developed stress fibres such as NIH 3T3 cells (Figure 2D). The actin binding protein vinculin is involved in linking the actin cytoskeleton with the extracellular matrix (ECM) and immunofluorescence staining of PhLP3 CHO LB01 knockdown cells for this protein revealed prominent focal adhesion in the elongated cells that appeared to be predominately at the cell periphery, in contrast to the staining pattern in control cells (Figure 2E).

### Rescue with human PhLP3 cDNA reverses or prevents morphological changes associated with RNAi mediated CHO LB01 cell PhLP3 silencing but disrupts mitosis and/or cytokinesis

To rescue the PhLP3 silenced phenotype, and confirm its cause as being due to PhLP3 depletion/knockdown, CHO LB01 cells were co-transfected with mouse PhLP3 siRNA and human PhLP3 cDNA. The basis of this approach is that the murine siRNA used in this study targets the 3′UTR of the mouse mRNA (and the CHO 3′-UTR as shown and described above) at a position where there is no sequence homology to the human 3′-UTR. The murine siRNA should not therefore knockdown the recombinant human *PhPL3* mRNA upon over-expression of the human cDNA and thus expression of the recombinant human PhLP3 in the rescue experiment can be achieved in the presence of the murine siRNA. To limit the disruption of the microtubule network and increased cell death evident when cells were transfected with large amounts (2 µg) of plasmid encoding PhLP3 (Figure 1D and 1E), a smaller amount (500 ng) of plasmid was used for rescue. Figure 3 shows typical results. While the elongated cell shape was prevented or reversed (compare 3C and 3D) by human PhLP3 expression on top of mouse siRNA, many cells either displayed enlarged nuclei or showed defects in late stages of mitosis compared to control transfections suggesting that the microtubule network was disrupted, if more mildly, as it is just by over-expressing PhLP3. The only mitotic stage observed in ‘rescued’ cells was either early or late anaphase which suggests that either progression through this stage in mitosis was disrupted by suprastoichiometric PhLP3 levels or that cytokinesis was inhibited or delayed, in agreement with spindle pole defects and aberrant cytokinesis observed during PhLP3 disruption in *C. elegans* [8].

### The RhoA/ROCK pathway is activated during actin stress fiber formation in PhLP3 siRNA silenced CHO LB01 cells

**Figure 4:**
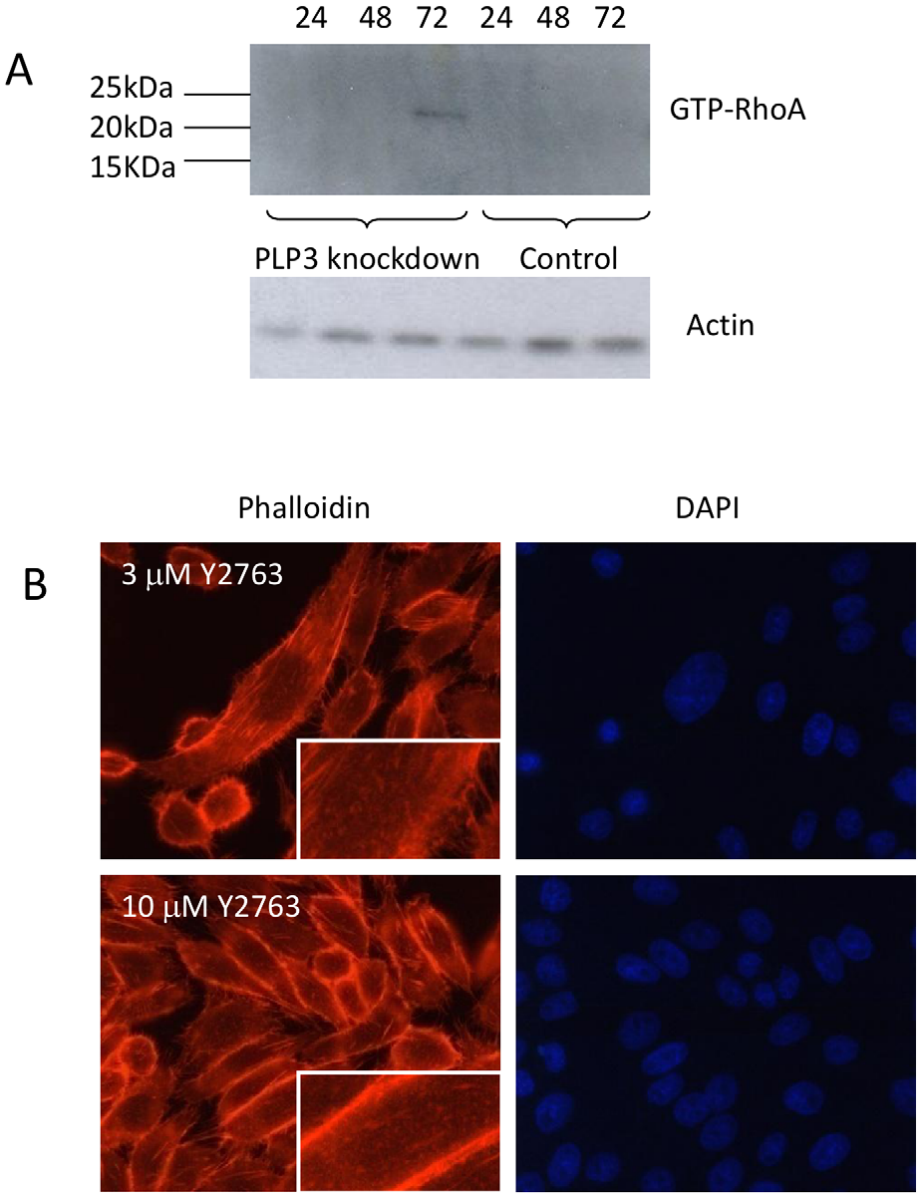
RhoA/p160ROCK pathway regulates actin stress fiber formation in PhLP3 siRNA silenced CHO LB01 cells. A commercially available RhoA-GTP/Rhotekin pull down assay was carried out on siRNA silenced PhLP3 LB01 cells. Samples were collected 24 h, 48 h and 72 h post transfection and activated RhoA was detected at 72 h post-knockdown with an anti-Rho A antibody but not in plasmid only control cells (A). LB01 cells transfected as in panel A for 72 h were then treated for 24 h with the ROCK inhibitor Y2763 (either 3 µM or 10 µM), fixed and stained for actin (rhodamine phalloidin, red). F-actin disassembly was observed at both Y2763 concentrations and the concentration of peripheral actin was observed with 10 µM Y2763 (B).

The small GTPase Rho A protein (RhoA) has a critical role in regulating cytoskeletal organisation which includes the formation of focal adhesions and actin stress fibers in cultured cells [32]. The rho-dependent serine threonine kinase p160ROCK has been identified as the major target for Rho [33] and in cultured cells, the pyrimidine derivative Y-27632 has been shown to specifically inhibit ROCK-mediated formation of stress fibers [34]. We evaluated whether this signalling pathway contributed to the reorganisation of the actin cytoskeleton observed upon PhLP3 silencing. In order to determine this, siRNA mediated knockdown of PhLP3 in LB01 cells was carried out over a period of 72 h and cells were collected at 24 h intervals for analysis of activated RhoA. Activated RhoA (24 kDa) was most strongly detected at 72 hours (Figure 4A), the time point coinciding with the most dramatic PhLP3 knockdown-dependent changes in cell morphology. To investigate RhoA involvement further, PhLP3 silenced LB01 cells were treated with either 3 µM or 10 µM of the Rho inhibitor Y-27632 for 6 h (namely during 72–78 h of silencing). This did not revert the elongated cell morphology, but did produce a discernible reorganisation of the actin cytoskeleton characterised by the substantial loss of the internal stress fibres, a concentration of actin filaments to the cell periphery and an increased number of filipodia compared to untreated cells (Figure 4B). These findings are consistent therefore with the hypothesis that the formation and stabilisation of the stress fibers observed upon PhLP3 silencing is RhoA dependent.

### Manipulation of PhLP3 levels in CHO LB01 cells is associated with altered MAPK phosphorylation

**Figure 5:**
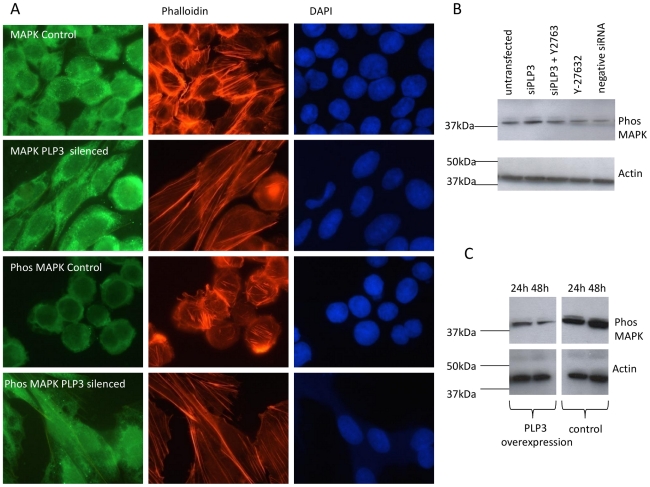
siRNA silencing of PhLP3 promotes MAPK phosphorylation upstream of RhoA activation and PhLP3 overexpression decreases levels of phosphorylated MAPK in suspension CHO LB01 cells. (A) PhLP3 silenced LB01 cells were fixed and co-stained with either anti-MAPK (second panel, green) and rhodamine phalloidin (for filamentous actin, red) or anti-phosphorylated MAPK (fourth panel, green) and rhodamine phalloidin. DAPI was used to detect DNA. Untransfected cells were used as controls (first and third panel). (B) An increase in phosphorylated MAPK was detected by immunoblotting using an anti-MAPK kinase antibody in cells transfected only with PhLP3 siRNA (lane 2) compared to cells that had been transfected with PhLP3 siRNA in the presence of the ROCK inhibitor Y2763 (lane 3). Cells treated with either Y2763 only, cells transfected with a ‘scrambled’ negative control siRNA and untransfected cells did not show elevated phosphorylated MAPK levels. (C) CHO cells were transiently transfected with PhLP3 for either 24 h or 48 h and levels of phosphorylated MAPK were detected by immunoblotting with an anti-MAPK kinase antibody. Expression levels of phosphorylated MAPK were found to decrease from 24 h to 48 h (compare lanes 1 to 2) and to be lower than in untransfected cells (lanes 3 and 4).

The dramatic change in morphology observed upon RNA silencing of PhLP3 in CHO LB01 cells is associated with the disassembly and reassembly of focal adhesions (Figure 2E). MAPK (ERK1/2) has an established role in focal adhesion disassembly involving calpain-activated cleavage of Rho and cytoskeletal linker proteins such as talin [35] but more recently MAPK was shown to also be required for their assembly and maturation [36]. Therefore, the status of MAPK phosphorylation was investigated during manipulation of PhLP3 levels, by immunoblotting with parallel examination of actin network organisation (by phalloidin staining) and, by immunofluorescence, of total MAPK versus phosphorylated MAPK. Figure 5A shows that there was no obvious change in the amount or distribution of total MAPK kinase in PhLP3 silenced cells compared to control cells. However, the phosphorylated MAPK in control cells was concentrated around the cell periphery and modestly evident whereas in silenced cells there was both an increase in amounts of phosphorylated MAPK and a more uniformly cytoplasmic and even nuclear distribution. Immunoblotting revealed highest levels of phosphorylated ERK at 72 h silencing compared to controls and that this increase was reduced in the presence of the ROCK inhibitor Y27632 (Fig. 5B). Conversely, during transient over-expression of PhLP3 there was a decrease in phosphorylated ERK over 48 h compared to controls (Fig. 5C).

## Discussion

We believe the studies here to be the first detailed report of the effects of manipulating PhLP3 levels on mammalian cells. Our findings are indicative of effects both on microtubules (principally manifested when PhLP3 is in excess of its usual amounts) and on microfilaments (when it is diminished). This is not unexpected, given that PhLP3 is proposed to modulate the actions of CCT, the chaperone most implicated in, and required for the folding of, both tubulin and actin, the required components of microtubules and microfilaments. Even so, the results obtained do appear to signify that PhLP3 appears to be important in regulating both of these cytoskeletal components, not just one or the other to their mutual exclusion. Indeed, they imply that the balance of PhLP3 levels in mammalian cells is rather crucial for the maintenance of cell health and the balance of deployment (and functions) of cytoskeleton subunits in the cytoskeletal network.

PhLP3 interacts with CCT to form a ternary structure with tubulin and is important in the regulation of β-tubulin folding [4]. Lacefield and Solomon [37] carried out deletion studies in yeast which showed that while it is non-essential, class III PLP (yeast Plp1) protects these unicellular organisms from the toxic effects of excess β-tubulin [38] and they concluded that PhLP3 was involved in an early step in β-tubulin folding. We show here that in Chinese hamster ovary cell lines, the overexpression of PhLP3 has dramatic effects upon microtubule networks, disassembling the interphase microtubule array, mislocalising β-tubulin to nuclei and upregulating β-tubulin levels while downregulating α-tubulin, an imbalance likely to influence dimer assembly and certainly one that is toxic to CHO cells (Figure 1) as observed for such imbalances in yeast. We note too that human PhLP2A (PDCL3 or VIAF, see Table 1) is an inhibitor of apoptosis (IAP) interacting factor that plays a role in caspase activation during apoptosis [39] demonstrating that at least one of the PLPs plays an active role in cell death. Interestingly, it has been suggested that in *plp1Δ* yeast cells, aggregation of α-tubulin is a result of the absence of correctly folded β-tubulin so heterodimers cannot be formed [37]. Determining how this imbalance arises upon PhLP3 over-expression will be a key factor in determining the role of PhLP3 in tubulin biogenesis.

We present here in Figure 3 findings indicating that knockdown of PhLP3 (using siRNA derived from mouse sequence) is permissive of microtubule assembly without obvious defects and yet it promotes reorganisation of the actin microfilament cytoskeleton to produce a more elongated anchorage-dependent phenotype. This contrasts with PhLP3 knockdown studies in *C. elegans* and *A. thaliana* that noted aberrant microtubule architecture as well as abnormal cytokinesis that could signify affects on actin too [8], [40]. A microtubule-based phenotype did however return upon partial rescue of CHO PhLP3 knockdown using human PhLP3 encoding DNA (Figure 3) where defects in mitosis were indicated by the observation of enlarged multinuclear cells (polyploidy) or disrupted chromosomal segregation indicative of delays in early or late anaphase. It is of interest to note that a temperature-sensitive yeast Plp2 mutant has implicated this protein in G1/S phase cell cycle progression [41] and it is possible that PhLP3 too may play a role in regulating chromosome separation during mitosis in mammalian cells, either directly through a tubulin folding based mechanism, or as a regulator of CCT. As discussed above, interference of PhLP3 interaction with CCT might shift the normal balance of its functions for other clients, including those with known regulatory function in cell cycle progression, including cdc20, cdh1 and plk1 [42], [43] that between them help to govern the anaphase to metaphase transition, mitotic exit and entry into S-phase.

Deletion studies in yeast and *in vitro* suggest that PhLP3 has a negative effect on actin expression [4] seemingly confirmed here by the influence of PhLP3 knockdown. Willison and colleagues have shown that yeast PhLP3 (Plp1) appears to inhibit the binding of actin to CCT whereas Plp2 (the yeast PhLP2 orthologue) strongly stimulates CCT-mediated actin folding [2]. If type III PLPs (human PhLP3 and yeast Plp1) stimulate β-tubulin folding while type II PLPs (human PhLP2 and yeast Plp2) instead stimulate actin folding then changing their balance could impact both tubulin and actin folding in reciprocal manner. Thus, our findings here in mammalian cells of a ‘fibroblast-like’ anchorage-dependent morphology induced by siRNA silencing of PhLP3 in CHO cells could reflect a tipping of this balance, promoting a re-arrangement of the actin cytoskeleton driving the formation of RhoA activated actin stress fibers and focal adhesions. The RhoA effector ROCK, is involved in these cytoskeletal changes since the selective ROCK inhibitor, Y27632, was observed to disrupts these actin stress fibers (Figure 4). In addition, these changes in the cytoskeletal architecture are accompanied by an increase in phosphorylated MAPK, while over-expression of PhLP3 reduced MAPK phosphorylation (Figure 5). Recently it was shown that the MEK-MAPK signalling pathway is required for focal adhesion formation of cells adhering to fibronectin [36]. It therefore remains to be established whether PhLP3 directly influences the formation of RhoA activated stress fibers and focal adhesions and whether phosphorylation of MAPK is a direct or indirect consequence of PhLP3 siRNA silencing and formation of focal adhesions, i.e. whether such changes are causal or instead outcomes and whether they occur by a direct or indirect mechanism. We note too (as above) that any perturbation of CCT by manipulating one of its regulators, such as here using PhLP3, is likely to impact on its many other clients and their functions that may feedback less directly into cyctoskeletal changes and could also, or instead, include any function CCT might serve in the adoption of quarternary, as opposed to tertiary, structure by its clients including, for example, any role it may play in regulating *in vivo* assembly of actin [13]. It is indeed interesting in this context that knockdown of the CCTε subunit in mammalian cells destabilises holoCCT assembly, releases CCT subunits to bind the cytoskeleton as monomers or subassemblies and, especially, is reported to produce a remarkably similar actin reorganisation-associated, elongated cell morphology phenotype to that observed here upon PhLP3 knockdown [44].

In conclusion, we note that activities of PLPs are regulated by phosphorylation [45], further complicating the analysis of these important proteins and hence studies focused on expression levels alone are unlikely to unravel the complete mechanism by which manipulation of these proteins influences the cytoskeleton. Despite this, the data presented here collectively shows that cytoskeleton remodelling and adherent-dependent growth can be activated in the industrially relevant CHO cell line by manipulating PhLP3 levels. For the bioprocessing of CHO cell lines engineered to express recombinant proteins it is necessary to suspension adapt these cells for growth in largescale bioreactors. The work we present here suggests that the reorganisation of the cytoskeleton via the manipulation of PhLP3 levels could therefore be further investigated as a method by which such cells can be switched between adherent and suspension culture for bioprocessing.

## Materials and Methods

### Plasmid Construction

Full length *Homo sapiens* thioredoxin domain containing 9 mRNA (Acc no BC005968) was obtained as an IMAGE clone (4073821) from Geneservice Ltd (U.K.). The full length protein coding sequence was obtained by PCR using forward primer 5′ACGTACGGATCCATGGAAGCTGATGCATCTGTT3′ and as reverse primer either 5′GTACGTTCTAGACTAATCATCATCAGAGTCTGA3′ to obtain an untagged clone or 5′GTACGTTCTAGACTATCATCATCAGAGTCTGA3′ to obtain a clone with a C-terminal V5 tag. PCR products were digested with BamHI and XbaI and cloned into a modified version of the plasmid vector pcDNA3.1 V5His-TOPO (Invitrogen) from which the ‘T overhang cloning site’ was removed by cleavage with BstXI and re-ligation. GFP plasmid, pEGFP-N1, was obtained from Clontech.

### Cell Culture

The CHO derived suspension cell line LB01, engineered to secrete a recombinant monoclonal antibody [9], [10] and the adherent cell line CHOK1 [46] were kindly provided by Lonza Biologics plc (UK). LB01 cells were cultured in CD CHO, a chemically defined media (Invitrogen, UK) which was supplemented with 25 mM methionine sulfoximine (MSX). The cells were grown in 250 ml shake flasks (Corning Inc, USA) in 5% CO_2_ at 37°C, with shaking at 100 rpm and routinely subcultured every 4 days at 3×10^5^ cells/ml. CHOK1 adherent cells were cultured at 5% CO_2_ and 37°C in Dulbecco's modified Eagles medium (DMEM/F-12) (Invitrogen, UK), 10% (v/v) heat inactivated fetal calf serum (FCS) (Lonza), MEM non essential amino acids (Invitrogen, UK; 100 µM), L-glutamic acid and L-asparagine (400 µM), adenosine, cytidine, guanosine and uridine (20 µM), thymidine (10 µM) and 10 mM L-glutamine.

### RNA Silencing

Double stranded small interfering RNAs (siRNAs) that corresponded to mouse PhLP3 (Mm_Txndc9_5) and human PhLP3 (Hs_TXNDC9_3) were obtained from Qiagen, UK. For immunofluorescence studies, cells were seeded in 24 well plates at 4×10^4^ cells/well and for immunoblotting in six well plates at 2×10^5^ cells/well. LB01 On the day of transfection LB01 cells were plated out in Dulbecco's modified Eagles medium (DMEM/F-12) (Gibco UK), 10% (v/v) heat inactivated fetal calf serum (FCS) (Lonza), MEM non essential amino acids (100 µM), L-glutamic acid and L-asparagine (400 µM), adenosine, cytidine, guanosine and uridine (20 µM) and thymidine (10 µM) to allow them to adhere to the culture dish. CHOK1 adherent cells were cultured as described above. Cells were transfected with either 150 ng siRNA (for 24 well plates) or 37.5 ng siRNA (diluted into the appropriate culture media) and either 12 µl or 3 µl of Hiperfect transfection reagent respectively, according to the manufacturers' protocol (Qiagen, UK). Cells were then harvested post-siRNA transfections for analysis at the time points described in the figure legends. For PhLP3 RNAi rescue experiments, CHOK1 cells were co-transfected with 37.5 ng siRNA and 500 ng plasmid for transient over-expression of PhLP3 as described below using Hiperfect as the transfection reagent as described by the manufacturer.

### Primary Antibodies used for Immunoblotting and Immunofluorescence

The following antibodies were used and sourced as indicated; ab56600 (an anti-thioredoxin domain containing 9 (PhLP3)) mouse monoclonal antibody, Abcam, UK; V8012 (anti-V5 clone V5-10) mouse monoclonal antibody, Sigma UK; AF1230 (anti-human/rat/mouse ERK2 rabbit polyclonal antibody which also recognises ERK1, R&D Systems, UK); 4370 (anti-phospho-p44/42 MAPK (ERK1/2), New England Biolabs, UK); V9131 (anti-vinculin) mouse monoclonal, Sigma, UK. The CCTα and CCTβ subunit antibodies (rabbit polyclonal) were gifts from Dr M Smith, University of Kent, UK; AC-15, anti-β-actin mouse monoclonal antibody (Sigma, UK); TAT (anti-α tubulin mouse monoclonal antibody) and KMX, anti-β-tubulin mouse monoclonal antibodies were gifts from Professor K. Gull (University of Oxford, UK). The anti-GFP mouse monoclonal antibody 3E1 was a gift from Professor W. Gullick (University of Kent, UK).

### Transient DNA Transfections for the Over-Expression of PhLP3

For over-expression studies, the appropriate amount of expression vector (500 ng or 1 µg as described in the figure legend) was transfected into LB01 cells which had been seeded at 1×10^6^ cells/well in a 24 well plate using Fugene HD (Roche Diagnostics Ltd, UK) transfection reagent according to the manufacturer's instructions. CHOK1 adherent cells were seeded at either 4×10^4^ cells/well (for immunofluorescence) or 1×10^6^ cells/well (for immunoblotting) and transfected using Lipofectamine 2000 (Invitrogen, UK) according to the manufacturer's instructions.

### Immunoprecipitation

For immunoprecipitation studies, cells were seeded at 3×10^5^ cells/well in 24 well plates and transfected with either the PhLP3 or PhLP3V5 encoding plasmid constructs as described above. Twenty four hours post-transfection, the cells were washed 3× with 1 ml of PBS and then lysed in 20 mM HEPES, pH 7.2, 100 mM NaCl, 1% (v/v) Triton X-100, 50 mM sodium fluoride, 1 mM sodium vanadate, 10 mM sodium β-glycerphosphate and Complete Protease Inhibitor Cocktail (Roche, UK). For immunoprecipitation, 2 µg of antibody was added to 200 µg of lysed protein and incubated for 2 h on ice. 25 µl of Protein A Sepharose slurry (Sigma, P3391) was then incubated with shaking with the antibody/lysate solution for 1.5 h at 4°C. The Protein A beads were then washed 3× in PBS and then 20 µl of 2× Laemmli SDS-PAGE sample buffer added, the samples boiled for 2 min, centrifuged in a bench top centrifuge at 13,000 rpm (17900 rcf) for 1 min and the eluted protein analysed by SDS-PAGE.

### SDS-PAGE Sample Preparation

Suspension cells were pelleted for 5 min in a benchtop microcentrifuge and the supernatant removed. The pelleted cells were washed twice in cold PBS before ice cold lysis buffer (see above) was added and the cells left for 5 min on ice to lyse. Adherent cells were washed twice in ice cold PBS, prior to addition of lysis buffer and then scraped with a Fisherbrand cell scraper (Fisher Scientific, UK). The lysate was then centrifuged at 13,000 rpm (17900 rcf) for two minutes and stored at −80°C until required for SDS-PAGE analysis.

### SDS PAGE Electrophoresis and Immunoblotting

SDS-PAGE was used to analyse proteins on 4–20% polyacrylamide gels (BioRad Labs Inc, UK). Proteins were semi-dry blotted for 1 h at 12 V onto Immobilon PDVF membranes (Millipore, UK). Non-specific binding sites were blocked with 4% Marvel milk proteins diluted in PBS for 60 min at room temperature. Immunoblots were incubated with primary antibodies (see below), which had been diluted in PBS/0.1% Tween 20 at 4°C overnight. The immunoblots were washed 5×5 min in PBS/0.1% Tween 20, probed with either rabbit anti-mouse IgG antibody or goat anti-rabbit IgG antibody conjugated to HRP (Sigma, UK) for 1 h at room temperature and washed again. The immunoblots were visualised on Hyper Film using the commercially available ssECL detection kit (Amersham Biosciences, UK).

### Indirect Immunofluorescence

Cells were seeded onto coverslips at 2×10^5^ cells/well in a 24 well plate and after the appropriate treatment were washed 2× in PBS, fixed for 10 min with 4% (w/v) paraformaldehyde in PBS, permeabilised in 0.1% Triton X-100/PBS for 5 min at 4°C, washed with PBS and non-specific binding sites blocked with 3% BSA in PBS for 20 min at room temperature. After the addition of primary antibody (diluted in 3% BSA in PBS) for 1 h at room temperature, cells were washed with 3% BSA in PBS and either anti-mouse FITC or anti-mouse TRITC conjugated rabbit IgG antibody (Sigma, UK) diluted 1∶100 or anti-rabbit FITC conjugated goat IgG antibody diluted 1∶64 with 3% BSA in PBS was added and left for 1 h at room temperature in the dark. Filamentous actin was detected using Phalloidin Alexa Fluor 546 (Molecular Probes, UK) which was diluted 1∶80 and incubated with the cells for 30 min. GFP (green fluorescence protein) was detected with 3E1 (an anti-GFP mouse monoclonal antibody). The cells were then washed and coverslips mounted in Mowiol containing *p*-phenyldiamine (1 mg/ml) before being left overnight at 4°C prior to microscopy using a Leitz DMBR immunofluorescence microscope.

### Activated RhoA Binding Assay

Activation of RhoA was determined using a commercially available pull down assay based upon detecting the interaction of the active GTP-bound RhoA with the Rho-binding domain (RBD) of Rhotekin (RhoA Activation Assay Kit, STA-403-A, Cell Biolabs Inc, USA). For this assay, 2×10^5^ cells were seeded in 6 well plates and siRNA silencing of PhLP3 was carried out as described above. Cells were subsequently collected 24, 48 and 72 h post transfection, lysed in a buffer consisting of 25 mM HEPES, pH 7.5, 150 mM NaCl, 1% Nonidet-40, 10 mM MgCl_2,_ 1 mM EDTA and 2% glycerol, and stored at −80°C prior to analysis by immunblot with an anti-RhoA antibody as described in the manufacturer's protocol.

### Inhibition of ROCK with Y27632

The Rho-kinase (ROCK) specific inhibitor Y27632 (Sigma Aldrich, UK) was added either at 3 µM or 10 µM to LB01 cells in which PhLP3 had been silenced for 72 h (as described above). The cells were incubated at 37°C for 6 h and then processed for immunofluorescence whereby actin filaments were detected with Phalloidin Alexa Fluor 546 (Molecular Probes, UK) as described above in the immunofluorescence section.

### Densitometry

Image J public access software (http://rsbweb.nih.gov/ij/features.html) (a Java image processing programme) was used for densitometry measurements of bands in immunoblots as per the instructions with the software.

## Supporting Information

Figure S1 **siRNA silencing of PhLP3 with a murine siRNA results in the knockdown of PhLP3 mRNA levels in CHO LB01 cells 72 h post-transfection.** CHO LB01 cells were transfected with an siRNA to PhPL3 as described in the methods section and the levels of mRNA determined by qRT-PCR 72 h post-transfection using a Chromo4 Real-Time PCR Detection System (Bio-Rad Laboratories, Inc.). Total RNA was extracted 72 h post-transfection using the RNeasy extraction kit (Qiagen) according to the manufacturer's instructions. For qRT-PCR the following primers were used; Forward: 5′ agtggaaatttaatggagcca; Reverse: 5′ gtatttctttccttggatagtt and the following PCR conditions; 50°C for 10 min followed by 95°C for 5 min, then 95°C for 10 s and 58°C for 30 s, these last two steps being repeated 35 times. qRT-PCR was undertaken using a QuantiFast SYBR Green RT-PCR kit (Qiagen). Crossing point, quantification, and melting curve analysis was undertaken using the Opticon Monitor software (Bio-Rad Laboratories, Inc.) by normalization to four house keeping genes. Control = no knockdown, PhPL3 KD = samples transfected with PhPL3 murine siRNA. Error bars represent Standard Deviation from the mean (SD), n = 3 biological triplicates.
(TIF)
