# Supplementary figures and images for: Modulation of Phosducin-Like Protein 3 (PhLP3) Levels Promotes Cytoskeletal Remodelling in a MAPK and RhoA-Dependent Manner
Source: PLoS One. 2011 Dec 9;6(12):e28271. doi: 10.1371/journal.pone.0028271 (PMC3235111; doi:10.1371/journal.pone.0028271)

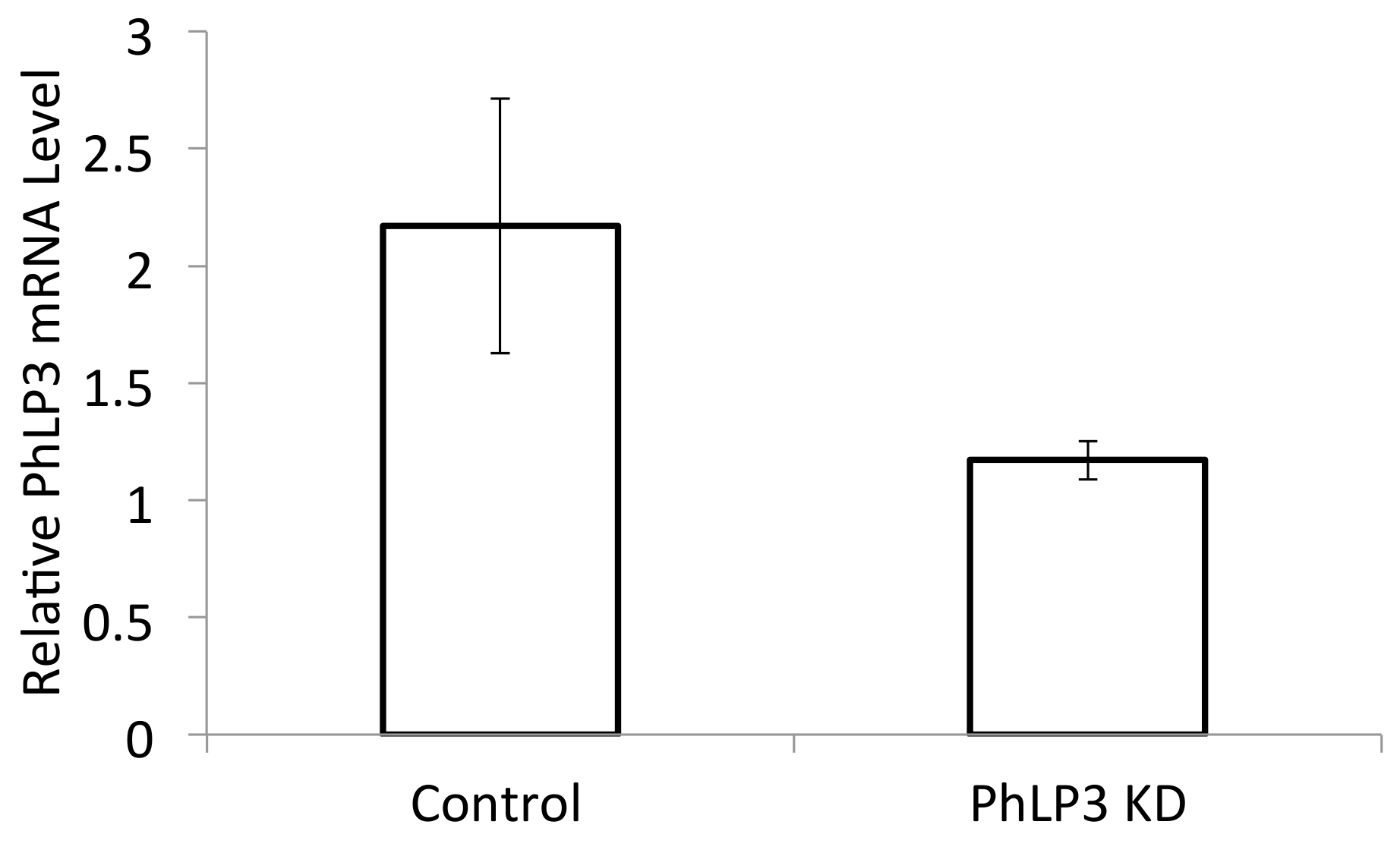

Supplement: Figure S1 — siRNA silencing of PhLP3 with a murine siRNA results in the knockdown of PhLP3 mRNA levels in CHO LB01 cells 72 h post-transfection. CHO LB01 cells were transfected with an siRNA to PhPL3 as described in the methods section and the levels of mRNA determined by qRT-PCR 72 h post-transfection using a Chromo4 Real-Time PCR Detection System (Bio-Rad Laboratories, Inc.). Total RNA was extracted 72 h post-transfection using the RNeasy extraction kit (Qiagen) according to the manufacturer's instructions. For qRT-PCR the following primers were used; Forward: 5′ agtggaaatttaatggagcca; Reverse: 5′ gtatttctttccttggatagtt and the following PCR conditions; 50°C for 10 min followed by 95°C for 5 min, then 95°C for 10 s and 58°C for 30 s, these last two steps being repeated 35 times. qRT-PCR was undertaken using a QuantiFast SYBR Green RT-PCR kit (Qiagen). Crossing point, quantification, and melting curve analysis was undertaken using the Opticon Monitor software (Bio-Rad Laboratories, Inc.) by normalization to four house keeping genes. Control = no knockdown, PhPL3 KD = samples transfected with PhPL3 murine siRNA. Error bars represent Standard Deviation from the mean (SD), n = 3 biological triplicates. (TIF) [file pone.0028271.s001.tif]
